# Pars Plana Vitrectomy with Internal Limiting Membrane Peeling for Refractory Diffuse Diabetic Macular Edema

**Published:** 2010-07

**Authors:** Mohammad-Hossein Dehghan, Masoud Salehipour, Jalil Naghib, Mahnaz Babaeian, Saeed Karimi, Mehdi yaseri

**Affiliations:** Ophthalmic Research Center, Labbafinejad Medical Center, Shahid Beheshti University of Medical Sciences, Tehran, Iran

**Keywords:** Pars Plana Vitrectomy, Internal Limiting Membrane, Macular Edema, Central Macular Thickness

## Abstract

**Purpose:**

To evaluate the effect of pars plana vitrectomy (PPV) with internal limiting membrane (ILM) peeling for management of refractory diffuse diabetic macular edema (DME).

**Methods:**

In this prospective interventional case series, eyes with refractory diffuse DME unresponsive to macular photocoagulation and/or intravitreal bevacizumab, and best corrected visual acuity (BCVA) ≥ 20/200 and ≤ 20/60 underwent triamcinolone-assisted PPV with ILM peeling. Pre- and postoperative evaluations included a complete ophthalmologic examination, fluorescein angiography and optical coherence tomography (OCT). Main outcome measures were BCVA and central macular thickness (CMT).

**Results:**

Twelve eyes of 12 patients with mean age of 59.6±3.9 (range, 55–68) years were operated and followed for a mean period of 4.9±1.0 (range, 4–6) months. Mean BCVA at final examination was 0.82 ± 0.18 logMAR which was not significantly better than its preoperative value of 1.00 ± 0.80 logMAR (P=0.959). Visual acuity improved by at least 2 lines in 3 eyes (25%), remained stable in 7 eyes (58%) and decreased by at least 2 lines in 2 eyes (17%). Mean CMT at final examination was 315±95 μm, which was significantly less than its preoperative value of 467±107 μm (P=0.004). Complications included vitreous hemorrhage in 2 and cataract progression in 5 eyes.

**Conclusion:**

PPV with ILM peeling for refractory diffuse DME seems to reduce macular thickness, but does not significantly improve visual acuity as observed after an intermediate-term follow up of about 6 months.

## INTRODUCTION

Macular edema is the leading cause of visual impairment in patients with diabetes mellitus.^1^ It progressively decreases visual acuity, with more than half of the patients losing at least 2 lines within 2 years.^2^ Diffuse diabetic macular edema (DME) is caused by extensive breakdown of the inner and outer blood-retina barrier, and its treatment is more challenging than that of focal edema which usually responds to laser macular photocoagulation (MPC) of microaneurysms. Several studies have shown that diffuse DME entails poor visual prognosis despite MPC.^3^–^6^

It has been suggested that after spontaneous detachment or surgical removal of the posterior hyaloid, tangential traction exerted by residual cortical vitreous and the internal limiting membrane (ILM) plays an important role in DME. Therefore there has been interest in pars plana deep vitrectomy (PPV) combined with ILM removal.^7^ Recent studies have suggested that PPV with ILM removal is effective for reducing or resolving DME and improving visual acuity.^7^–^14^

The purpose of this prospective study was to evaluate the effectiveness and safety of triamcinolone-assisted PPV together with ILM peeling in diffuse refractory DME unresponsive to MPC or intravitreal bevacizumab (IVB).

## METHODS

In this prospective interventional case series, consecutive eyes with diffuse DME refractory to MPC and/or IVB at Labbafinejad Medical Center, Tehran, Iran from September 2006 to September 2008 were evaluated for eligibility. Inclusion criteria were: 1- persistent diffuse DME defined as central macular thickness (CMT) ≥ 250μm with history of at least two sessions of MPC or IVB, or one session of each modality performed more than 4 months prior to PPV; and 2- best corrected visual acuity (BCVA) ≥ 20/200 and ≤ 20/60. Exclusion criteria were: 1- lens opacity precluding optical coherence tomography (OCT); 2- massive hard exudates in the fovea; 3- very severe non-proliferative diabetic retinopathy (NPDR) or any degree of proliferative diabetic retinopathy (PDR); 4- previous vitreoretinal surgery; 5- evidence of posterior vitreous detachment (PVD) and/or vitreomacular traction; 6- angiographic evidence of macular ischemia; 7- any concomitant ocular disease; and 8- monocular patients.

Preoperative evaluations included determination of Snellen BCVA, biomicroscopic examination of the anterior and posterior segments, fluorescein angiography (FA), and OCT (3D OCT-1000, Topcon Corporation, Tokyo, Japan) for measuring CMT.

Written informed consent was obtained from those who fulfilled the study criteria before proceeding with surgery. All eyes underwent standard 20-gauge three-port PPV. After core vitrectomy, induction of PVD was initiated on the nasal side of the optic disc using aspiration from a soft-tipped cannula or vitrector. After completion of vitrectomy, 0.1 ml (4 mg) of triamcinolone was injected over the macula with the infusion turned off and then washed out immediately with active suction. The ILM was incised with a bent MVR blade and peeled at a distance of at least one disc diameter from the fovea in all directions using intraocular forceps. No tamponade was used at the end of surgery.

Postoperatively, all patients were visited one day, one and four weeks after the procedure, and every 2 to 3 months thereafter. Chloramphenicol, betamethasone, and cycloplegic drops were started postoperatively and gradually tapered off within 4 weeks. At each follow up visit, Snellen BCVA was determined and a complete ophthalmologic examination was performed; OCT and FA were repeated every 3 months.

Cataract surgery was performed whenever the lens opacity was severe enough to preclude FA and/or OCT. Pre- and postoperative BCVA was converted to logarithm of minimum angle of resolution (logMAR) notations and compared by Wilcoxon signed-rank test. Pre- and postoperative CMT were also compared by Wilcoxon signed-rank test.

## RESULTS

Twelve eyes of 12 patients (6 male) were included in the study. Table 1 shows basic characteristics, and pre- and post-operative BCVA and CMT. Mean age of the patients was 59.6±3.9 (range, 55–68) years and all subjects had type 2 diabetes mellitus. Diabetic retinopathy was graded as severe NPDR in 6 eyes and as moderate NPDR in 6 other eyes. In all eyes, macular edema was appreciated biomicroscopically, and diffuse fluorescein leakage was visible angiographically. All eyes had received at least two sessions of MPC, and/or IVB. The mean intervals between the most recent MPC or IVB, and PPV were 7.1 (range, 5–14) and 5 (range, 4–9) months, respectively.

Mean BCVA at final follow-up was 0.82±0.18 logMAR which was not significantly better than its preoperative value of 1.00±0.80 logMAR (P=0.959). Visual acuity improved by at least 2 lines in 3 eyes (25%), remained stable in 7 eyes (58%) and decreased by at least 2 lines in 2 eyes (17%).

Mean CMT at final follow-up was 315±95 μm, which was significantly less than its preoperative value of 467±107 μm (Wilcoxon test, P=0.004). At final examination, CMT had decreased by more than 20% in 6 of 12 eyes (50%).

No major intraoperative complication developed in any of the patients. Two eyes developed PDR with vitreous hemorrhage (cases 1 and 3) during the follow-up period and required repeat deep vitrectomy. Case 3 developed neovascular glaucoma with poorly controlled intraocular pressure (IOP); this subject underwent cyclophotocoagulation and IOP was eventually controlled without any medications. Preoperatively, one eye was pseudophakic (case 2) while the others had mild or no lens opacity. After vitrectomy, progression of lens opacity occurred in 5 eyes, but no eye required cataract surgery up to 6 months of follow up. After 6 months, cases 1 and 3 underwent cataract surgery. Table 2 shows mean pre- and postoperative BCVA and CMT after excluding cases 1 and 3 who developed vitreous hemorrhage.

## DISCUSSION

DME remains a significant cause of visual impairment in developed countries. The cornerstone for treatment of DME is MPC, which has been shown to reduce the incidence of moderate visual loss in eyes with clinically significant macular edema by 50%.^2^ In eyes with diffuse DME, however, the visual response to MPC is less encouraging.^15^ Lee and Olk^6^ in their series of 302 eyes with diffuse DME treated by MPC, reported visual improvement equivalent to three Early Treatment of Diabetic Retinopathy Study (ETDRS) chart lines or more in only 13.7% of eyes at one year, and visual decline in 25% after three years. Visual rehabilitation for this subset of patients, who do not respond to laser photocoagulation, presents a significant challenge. The biological plausibility of vitrectomy for DME has been suggested by clinical observations that PVD is associated with a lower incidence of DME. In a retrospective study by Nasrallah et al^16^ only 20% of eyes with DME had PVD, whereas 55% of eyes without DME had PVD. Vitrectomy has been shown to be efficacious in eyes with identifiable hyaloid thickening or contraction, but results in eyes without hyaloidal abnormalities are conflicting.

In this prospective interventional case series of patients with refractory diffuse DME treated by PPV combined with ILM peeling, we observed a significant improvement in foveal thickness. However, visual acuity improvement was not significant, even after excluding 2 eyes complicated by postoperative vitreous hemorrhage. Visual acuity improvement by two or more lines has been reported in 43 to 92% of eyes undergoing PPV with ILM removal.^7^–^11^,^17^ There are reports that PPV with ILM peeling reduces retinal thickness without improvement in vision.^18^–^20^ In our study, the percentage of eyes that experienced at least 2 lines of improvement (25%), was less than that of the above-mentioned studies^7^–^11^,^17^. Such discrepancies may be due to differences in: 1- duration of macular edema prior to surgery; 2- prior MPC and/or IVB; 3- severity of diabetic retinopathy; 4- severity of macular ischemia which cannot always be detected on FA; and 5- duration of follow up.

There is agreement in nearly all previous studies that PPV with ILM peeling significantly reduces macular thickness,^7^–^12^,^14^ although this is not exactly correlated with improvement in visual acuity,^18^–^20^ as has been the case in our study. The disparity between anatomic and visual outcomes may be due to both an increase in lens opacity and worsening of diabetic retinopathy.

The role of ILM removal in DME is unclear. Some investigators have reported PPV without ILM removal to be as effective as surgery with ILM removal in terms of reduction in retinal thickness and improvement in visual acuity.^12^,^21^ Conversely, a retrospective study reported that PPV effectively reduced DME, but results were better in eyes with ILM removal than those without ILM removal.^17^ Gandorfer et al^7^ observed that diffuse macular edema which progresses despite PPV together with posterior hyaloid and epiretinal membrane removal for non-clearing vitreous hemorrhage, resolves rapidly after ILM removal. Kimura et al^14^ also described prompt resolution of diabetic cystoid macular edema in eyes that had undergone previous PPV without an epiretinal membrane. Residual cortical vitreous has been demonstrated to remain attached to the macula after removal of the posterior hyaloid during triamcinolone-assisted PPV.^22^ Internal limiting membrane thickening and cell abundance on the vitreous side of the ILM has been observed in eyes with diabetic maculopathy.^23^ Therefore, ILM peeling may have a beneficial effect in DME by removing the tangential traction exerted by the ILM and residual cortical vitreous. Furthermore, ILM removal may also have a beneficial effect in preventing postoperative epiretinal membrane formation by removing the scaffold for proliferating cells. Postoperative epiretinal membrane formation, which has been described in 10.2% to 13.8% of eyes after PPV without ILM peeling,^24^–^26^ was not observed in our study and other similar studies utilizing PPV with ILM peeling,^7^–^12^ except for one study which reported epiretinal membrane formation in 5% of eyes.^14^

Postoperative complications in our study included progression of diabetic retinopathy from NPDR to PDR with vitreous hemorrhage in 2 eyes and neovascular glaucoma in one other eye; the latter complication may not have been related to deep vitrectomy. Progression in lens opacity which occurred in 5 eyes is a common finding after vitrectomy.

Of the limitations of the current study is the possible edema-reducing effect of vitrectomy, removal of the posterior hyaloids, and triamcinolone. During the procedures, we attempted to completely wash out the injected triamcinolone, so that its effect would be negligible. Moreover, systemic factors, especially HbA_1c_ and lipid levels, may have acted as confounding factors but were not evaluated in our study.

In conclusion, vitrectomy with ILM peeling for refractory diffuse DME may reduce macular thickness, but does not seem to significantly improve visual acuity during intermediate-term follow up of about 6 months. A large multicenter randomized controlled trial comparing PPV with and without ILM peeling with longer follow up is required to establish the role of ILM removal for treatment of DME.

## Figures and Tables

**Table 1:** Demographic information, preoperative and postoperative visual acuity and central macular thickness in eyes with refractory diffuse diabetic macular edema

Case	Age (years)	Sex	Eye	Grade of NPDR	F/U (Months)	BCVA (logMAR)		CMT (μm)	
						Baseline	Final	Baseline	Final
1	62	M	OS	Severe	6	20/200	HM	503	-
2	60	M	OS	Severe	6	20/120	20/160	276	248
3	60	M	OD	Severe	6	20/120	HM	455	392
4	68	F	OS	Moderate	6	20/200	20/160	564	409
5	65	F	OS	Moderate	4	20/200	20/25	554	276
6	55	F	OD	Severe	4	20/60	20/60	591	252
7	60	M	OD	Severe	4	20/160	20/100	341	215
8	62	F	OD	Moderate	4	20/200	20/320	623	228
9	58	M	OS	Moderate	4	20/80	20/80	420	359
10	60	F	OS	Severe	4	20/160	20/200	452	467
11	58	M	OD	Moderate	5	20/80	20/50	485	409
12	55	F	OD	Moderate	6	20/120	20/100	357	191

NPDR, non-proliferative diabetic retinopathy; F/U, follow-up; BCVA, best corrected visual acuity; CMT, central macular thickness; M, male; F, female; OS, left eye; OD, right eye; HM, hand motions

**Table 2:** Pre- and postoperative mean BCVA and CMT excluding cases 1 and 3 who developed postoperative vitreous hemorrhage

	Preoperative		Postoperative		Mean change	P value

	Mean±SD	Range	Mean±SD	Range		
BCVA (logMAR)	0.80±0.19	0.48–1	0.68±0.31	0.0–1.1	0.13±0.30	P=0.159
CMT (μm)	468±107	276–623	307±97	191–467	159±135	P=0.007

SD, standard deviation; BCVA, best corrected visual acuity; CMT, central macular thickness
